# The do’s, don’t and don’t knows of supporting transition to more independent practice

**DOI:** 10.1007/s40037-018-0403-3

**Published:** 2018-01-30

**Authors:** Sarah Yardley, Michiel Westerman, Maggie Bartlett, J Mark Walton, Julie Smith, Ed Peile

**Affiliations:** 1 Central and North West London NHS Foundation Trust, London, UK; 2 VU Medical Center Amsterdam, Amsterdam, The Netherlands; 3 Dundee School of Medicine, Dundee, UK; 4 McMaster Children’s Hospital, Hamilton, Ontario Canada; 5 Nuffield Orthopaedic Centre, Oxford, UK; 6 St Catherine’s College, Oxford, UK

**Keywords:** Careers, Education, Medical, Transition, Physicians, Progressive independence

## Abstract

**Introduction:**

Transitions are traditionally viewed as challenging for clinicians. Throughout medical career pathways, clinicians need to successfully navigate successive transitions as they become progressively more independent practitioners. In these guidelines, we aim to synthesize the evidence from the literature to provide guidance for supporting clinicians in their development of independence, and highlight areas for further research.

**Methods:**

Drawing upon D3 method guidance, four key themes universal to medical career transitions and progressive independence were identified by all authors through discussion and consensus from our own experience and expertise: workplace learning, independence and responsibility, mentoring and coaching, and patient perspectives. A scoping review of the literature was conducted using Medline database searches in addition to the authors’ personal archives and reference snowballing searches.

**Results:**

387 articles were identified and screened. 210 were excluded as not relevant to medical transitions (50 at title screen; 160 at abstract screen). 177 full-text articles were assessed for eligibility; a further 107 were rejected (97 did not include career transitions in their study design; 10 were review articles; the primary references of these were screened for inclusion). 70 articles were included of which 60 provided extractable data for the final qualitative synthesis. Across the four key themes, seven do’s, two don’ts and seven don’t knows were identified, and the strength of evidence was graded for each of these recommendations.

**Conclusion:**

The two strongest messages arising from current literature are first, transitions should not be viewed as one moment in time: career trajectories are a continuum with valuable opportunities for personal and professional development throughout. Second, learning needs to be embedded in practice and learners provided with authentic and meaningful learning opportunities. In this paper, we propose evidence-based guidelines aimed at facilitating such transitions through the fostering of progressive independence.

## Definitions of do’s, don’t and don’t knows

### Do’s

Educational activity for which there is evidence of efficacy

### Don’ts

Educational activity for which there is evidence of no efficacy or of harms (negative effects)

### Don’t knows

Educational activity for which there is no evidence of efficacy or of harms (negative effects)

## Introduction

### Background

Learners need to successfully navigate several transitions within the medical education trajectory prior to delivering unsupervised medical practice as consultants or general practitioners. [1] Transitions within medical education are regularly portrayed as daunting and existing literature reports on burnout and depression among postgraduate trainees. [2] Several papers report that these negative emotional outcomes, i. e. burnout, depression or anxiety, pertain to a sudden increase in level of responsibility within patient care provision. [3–10] Changes in responsibility occur throughout the transition to ‘independent’ practice. [5, 11–14] We postulate that a better implementation of progressive independence [14] within medical training aids the successful transition to unsupervised practice. [15] Therefore, by presenting these guidelines we aim, first, to bring together evidence-based understanding of fostering transitions in progressive independence and, second, to identify what remains unknown and in need of further research. This is the third D3 paper in this series [16]: guidelines are formulated as practice *Do’s, Don’ts and Don’t knows*. Since many clinicians find themselves simultaneously in transition and providing support to those within transition, our aim is to provide helpful insights or guidelines for both these transition positions. *Do’s *describe what should be enacted based on existing empirical evidence. *Don’ts* describe what should better be avoided based on evidence it does not work or may cause harm and *Don’t knows *describe concepts or interventions about which there is genuine equipoise or uncertainty about effect [16].

We provide a brief summary of key evidence to support our recommendations, indicating the strength of evidence along the way (see Table 1 for the criteria). This summary is based on a scoping review of the literature regarding transitions in medical practice combined with our own experience and study of this area. Table 2 shows a list of the Do’s, Don’ts and Don’t knows.

**Table 1:** Criteria for strength of recommendation

Strong	A large and consistent body of evidence
Moderate	Solid empiric evidence from one or more papers plus the consensus of the authors
Tentative	Limited empirical evidence plus the consensus of the authors

**Table 2:** Summary of guidelines for clinicians experiencing transition and those providing support

**Transitions to independent practice: workplace learning**		Strength of recommendation
**Do’s**	Embed learning in practice and provide authentic and meaningful learning opportunities	Strong
**Don’ts**	Avoid rather than manage risk	Moderate
**Don’t knows**	What is the best balance between prescriptive programs tailored to individuals and ‘one size fits all’ competency-based or target-based training programs?	–
**Transitions to independent practice: independence and responsibility**		
**Do’s**	Provide meaningful pre-transition preparation courses linked to local inductions	Moderate
	Encourage progressive independence by offering a sliding scale of decreasing supervision alongside demonstrating increasing trust (both globally and for specific tasks)	Moderate
	Apply the concepts of graduated responsibility to non-clinical as well as clinical domains of training, such as leadership and responsibility	Moderate
	Make postgraduate trainees aware of the psychological impact of actual responsibility (including the process of their own identity formation) once they move up a level of training or into consultancy	Moderate
**Don’ts**	Treat transition as a moment in time	Strong
**Don’t knows**	What is the best way to ensure ongoing continuing professional development as a consultant or general practitioner?	–
	Can we move understanding of the outcomes and impact of transition beyond perceptions?	
	What is best practice in helping doctors-in-difficulty or training-departments-in-difficulty? In optimizing transitions, what should be the role of regulatory bodies and employers?	
**Transitions to independent practice: mentoring & coaching support**		
**Do’s**	Establish a mentorship program with local champions	Moderate
	Seek to aid the development of resilience and independence	Moderate
**Don’t knows**	How do we develop common understanding around what is optimal in mentoring and coaching for multidimensional medical roles?	–
**Transitions to independent practice: patient perspectives**		
**Don’t knows**	How can patient feedback encourage effective transitions to independent practice and contribute to risk management?	–
	What is the best way to manage the tension between creating opportunities for progressive clinical independence as a learning mechanism and managing patient safety?	

### Definitions

The trajectory towards unsupervised medical practice covered starts with evidence regarding the move from undergraduate to postgraduate (‘becoming a qualified doctor’) to general postgraduate and specialist training (residency) and continues into the first few years of practice as a consultant (attending physician). In this paper, we have used the term consultant rather than attending physician throughout as this was commonest within the literature. When the evidence allows, we distinguish between undergraduates (students), postgraduate trainees (including but not limited to residents with the more precise term used when applicable), consultants and general practitioners or particular medical specialties. The generic term doctor is used alone when this is not possible. ‘Independent practice’ is an increasingly controversial term; in this work, we have used it when appropriate, and in line with the original research discussed, to mean capable of unsupervised practice acting with an appropriate measure of independence. It does not imply reaching a point of individual autonomy that precludes appropriate collaborative working and co- or inter-dependence for continuing professional development.

## Methods

In order to come up with practical suggestions to foster positive transitions, we have limited our remit to evidence specifically related to transitions, drawing on the D3 method guidance received [17]. First, we identified themes and principles applying throughout career trajectories across the whole of medical practice through discussion and consensus, with each author bringing their existing knowledge and expertise in the field. This resulted in all authors reaching consensus on four key domains (overarching themes) for inclusion in our work: workplace learning; independence & responsibility; mentoring & coaching; and patient perspectives. Subdivisions among themes were subsequently refined as we conducted a scoping review of the literature (see below) and we remained sensitive to the possibility of new additional domains but none were found. Therefore we collectively organized identified literature results and extractable principles into the above domains (iteratively refining these in light of the evidence). As the review progressed we moved from tentative to agreed subdivisions of evidence in each domain as Do’s, Don’ts and Don’t knows. As we set out to report the domains considered most important for, and specific to, transitions towards independent practice we present our findings under each of these domains, despite variations in the volume and quality of existing literature.

The purpose of this review is to evaluate the evidence clinical teachers and postgraduate trainees can apply to managing transitions to independent practice, rather than to document the well-recognized need for this to be better supported. Therefore, we did not include studies that focussed solely on organizational or structural anticipatory preparations of preparedness for a change in status without touching upon the lived experience of progressive independence. Exclusions were also made when the focus of a study was to quantify need or was purely structural, such as designing curricula to include taught skills prior to practical application in authentic scenarios, since these findings are hard to translate into practical Do’s, Don’ts and Don’t knows. Many other studies initially identified in our scoping were limited to narrowly defined geographical, institutional or specialty specific issues and were therefore excluded unless we were able to identify elements of more transferable data.

**Fig. 1:**
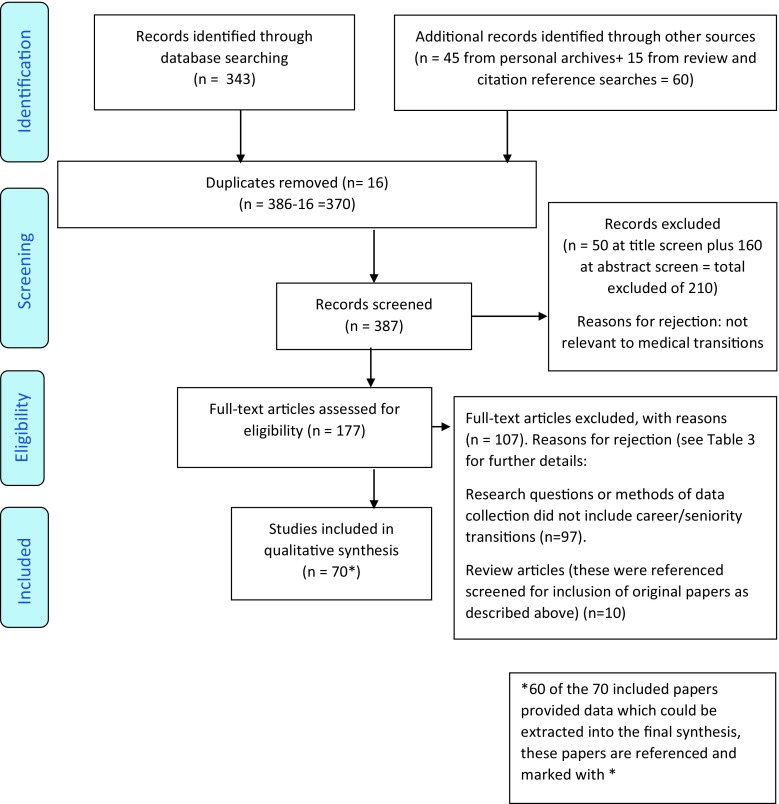
PRISMA flow diagram

We used the principles advocated by Arksey and O’Malley [18] for our scoping review. We also drew on the previous methods of reviewing transition literature used by Teunissen and Westerman [19]. We first pooled the relevant literature from our personal archives, each contributing literature from our experience of the scholarship of transitions. Then we conducted scoping searches as described in Table 3 and Fig. 1. Although we had identified a priori domains of importance we did not use these as parameters or exclusion reasons during the scoping searches in order to prevent loss of any additional evidence specific to transitions. All included articles were reference screened.

**Table 3:** Scoping searches

1. Medline database search using the search string <[Transition OR trajectory] AND Medical Education [limit] review articles. Identified 81 articles which were screened for relevance and those selected were then reference screened for additional original work to add to the records generated from personal experience of the literature. A total of 44 papers remained eligible from these searches after full-text screening
2. Medline search using the search string <[Transition OR trajectory] AND [Professional Autonomy [exp MeSH] OR independen* OR Professional Competence [MeSH] OR Clinical Competence [MeSH] OR [EPA or Entrusted professional activity] OR Mentors [MeSH] OR coach* OR supervis* OR social support OR pastoral care OR Prepar* OR stage OR progress* OR [CCT or completion of certificate in training] OR board certificat* OR Workplace learning OR practice-based learning OR Clinical reasoning OR decision making OR Rehears* OR Resilience, Psychological [MeSH]]. This was limited to specific journals as the initial search was not sensitive enough. Selected journals were Medical Education, Academic Medicine, Medical Teacher, Advances in Health Sciences and Education, BMC Medical Education, Teaching and Learning in Medicine, Perspectives on Medical Education and BMJ. 262 articles were identified through this wider search string. Following the removal of duplicates on combining references from this search string with our existing records and addition of citation checks the total for screening was 387 papers
3. Of the papers screened, 210 were excluded at title or abstract screening and a further 107 at full text screening. All authors contributed to screening, with at least two authors screening each record at each stage
*Reasons for rejection were:*
– Not relevant to medical transitions—evident from title/abstract screening (*n* = 210)
– Research questions or methods of data collection did not include career/seniority transitions (*n* = 97).
*Examples of articles excluded on this basis included those:*
– Focused on specialty selection/assessment rather than student/trainee/consultant transitions testing methods of assessment or teaching specific tasks/skills separate to transitions in clinical practice
– About organizational or institutional transition to new structures, or technological innovations for clinical practice
– Historical articles on introduction of problem-based learning in undergraduate settings
– Transition of international graduates into Western healthcare employment
– Hierarchies and transitions in non-clinical careers
– Only about preparation rather than the actual transition
– Local and specialty specific surveys if findings not more widely relevant or potentially transferable
– Curriculum design and evaluation that is not actually about transition to independent practice
– Impact of transitions in demographic make-up of medical graduates
Review articles (these were reference screened for inclusion of original papers as described above) (*n* = 10)

We did not pre-specify types of interventions, comparisons or outcomes in our searches, or pre-set quality criteria as we wanted to identify the breadth, scope and quality of existing literature. No non-English articles were identified in our searches

For the analysis we used a data extraction sheet (see Appendix) to identify key elements of each paper according to our chosen themes and to allow assessment of quality. The data extraction was divided between JMW, MB, JS and SY with SY reviewing all records. A narrative synthesis using both the original papers and our data extraction sheets was generated with different authors leading on different themes but with all contributing to the overall synthesis and judgement regarding quality of evidence for each theme.

Our scope of the literature yielded 387 articles which were identified and screened. Of these, 210 were excluded for not being relevant to medical transitions (50 at title screen; 160 at abstract screen). A total of 177 full-text articles were assessed for eligibility; a further 107 were rejected (97 did not include career transitions in their study design; 10 were review articles—the primary references of these were screened for inclusion and when relevant used as a source of primary evidence).

Seventy articles were included of which 60 provided extractable data for the final qualitative synthesis. Across the four key themes, seven do’s, two don’ts and seven don’t knows were identified along with the strength of the evidence for each. We will describe the above-mentioned domains, and for each domain we start with do’s, then don’ts, and finish with don’t knows in the results section below.

In addition to reference screening the ten review articles identified, we used these reviews to guide our work. Teunissen and Westerman [19] conducted a review of the transition literature some years prior to our current work. As well as drawing on their methods, we used their review to develop the themes investigated, in particular, to orientate us to investigate further issues of independence and responsibility. These issues were identified by Kennedy et al. [15] who argue for a model of progressive independence. These authors recognized that further empirical work was needed to test the theory of such a model in practice. Abernethy et al. [20] describe the challenges of ensuring adequate yet efficient surgical training, noting the pressure to reduce time to develop expertise. Taherian and Shekarchian [21], and Sambunjak et al. [22] also reported the strength of perceptions that good mentoring was perceived as highly beneficial for physicians although it was not well defined and the essential elements for effectiveness not completely understood. Nonetheless, this finding was confirmed in the later review of mentoring conducted by Entezami et al. [23] Other relevant areas in which definitions and understanding have been reviewed include competency-based education [24, 25] and preparation for practice. Cameron et al. [26] provide a useful overview of the literature on preparation of newly qualified doctors for practice. In addition to identifying contextual factors, the nine studies in their review described anxieties particularly with respect to prescribing and practical procedures and/or personal traits that impact perceptions of preparedness. This review also highlighted the need for further prospective studies to identify what makes a difference to the experience of transition. Most recently, Monrouxe et al. [27] identified that much of the literature on preparedness suffers from focusing too narrowly on pre-graduation and on very short time periods post-graduation. Previous insights are mainly limited to perceptions rather than other measures of impact, and lacked depth of understanding with respect to what, why, and how is most efficient and effective. This also resonates with the fact that medical education literature tends to frame transitions as short periods of time rather than as prolonged developmental processes.

## Results

### Workplace learning

#### Do’s

##### **Guideline 1.**

*Embed learning in practice and provide authentic and meaningful learning opportunities (* **strong** *)*

The provision of learning opportunities that include authentic (‘real life’) scenarios with collaborative and relevant learning goals is theoretically sound [28]. Experiential learning theories suggest that authentic learning becomes increasingly important as learners become more independent. In transitioning to independent practice, doctors require repeated experiences to build on their understanding of principles [1]. Empirical work has now established that workplace-based learning goes hand-in-hand with practice, in both simulated healthcare [29, 30] and clinical settings [31–33]. Readiness for increasingly independent practice depends upon having opportunities for learning ‘on-the-job’. Opportunities should include authentic part and whole tasks with appropriate supervision to develop technical and non-technical skills without compromising patient safety [29–33]. Authentic complex training scenarios with debriefing should be provided alongside meaningful real time entrustable professional activities [34] and programmatic assessment [35] which includes a strong formative element.

Transitions have been reframed as critical intensive learning periods (CILPS) in which a learner’s performance is critically dependent upon the local working environment [36–38]. Learning experiences are meaningful when ‘practical know-how’ is gained in practice. It is perhaps because of this that sufficient workplace learning has been identified as the major challenge in the transition from undergraduate to postgraduate and in the transition from trainee to consultant. Of these, undergraduate to postgraduate is also by far the most researched, with only a small emergent literature about transition from trainee to consultant and even less regarding progressive transitions within career grades or continuing professional development. Hence less is known about what the challenges might be in these transition periods.

Lack of practical know-how leads to generalised feelings of uncertainty and ambivalence in seeking support [4, 6]. It may be that this is due to the tension between wanting to prove oneself at a new level of responsibility while experiencing lack of confidence (or competence) for practice at this new level. Some research suggests that this tension may be compounded by the tendency of induction experiences to focus on the organizational and institutional agendas rather than providing meaningful induction into the day to day working of the local setting and role [7, 9].

Kilminster et al. [36] found that actual practice was determined much more by situational and contextual factors than by regulatory and organizational frameworks. Practice varies upon the clinical setting, the healthcare provider, shift type (day/night), composition of the medical team and the presence of other healthcare professionals [36]. Changes in training programs, the organization of healthcare, and anxieties about patient safety (see below), have led to concerns that it is harder for postgraduate trainees to get appropriate exposure to certain clinical scenarios and to gain enough technical skills practice [39]. Together these changes led to it being ever more important that workplace-based clinical teachers and supervisors help postgraduate trainees to access necessary learning opportunities.

Decision-making, initiative-taking, prioritization skills and coping with stress are important components in preparedness and transition [40]. Supervisors require a range of skills in assessment of learners in order to avoid reductionism and to take a global, holistic view of the learner [41, 42]. Illing et al. [31] and Jones et al. [32] recommend increased on-the-job opportunities while Ker et al. [29, 30] identified the role for realistic simulated ward exercises in preparedness for practice. Specific attention to acute care and prescribing has been highlighted within actual clinical practice [31, 40, 43–45] while Lempp et al. [44] specified the importance of continuity of care and advanced communication skills.

#### Don’ts

##### **Guideline 2.**

*Avoid rather than manage risk (* **moderate** *)*

Risk avoidance in the workplace setting may hamper effective learner transition. Instead of developing their ability to handle change and learn from experiences, they may be merely trained to work well at their current level, without increasing independence [46]. De Feijter et al. [47] found that interpersonal relationships and trust between supervisor and learner were key to appropriate levels of supervision to protect patient safety while Kennedy et al. [46] found fluidity between different types (and supervisor interpretations) of ‘clinical oversight’ (routine/responsive/backstage). The point at which a supervisor permits movement from any of these types of oversight into direct patient care is not purely trainee competence dependent, but subject to the supervisor’s own pedagogical stance.

A number of organizational risks that can impede learners’ transitions have also been identified: rota issues, varying quality of induction into new workplaces and roles, multiple transitions within rotations [9, 36], new work patterns, and inadequate staffing [44]. Unnecessary stress is detrimental to both learners’ performance and the quality of patient care during transitions, which are already stressful because of increased responsibility, uncertainty and the need to re-form interpersonal relationships [4, 6, 38]. Within the surgical context, the lack of opportunity to operate autonomously is detrimental to the development of competence and transition to independent practice [39], yet inadequate supervision is detrimental to skill development and patient safety [48].

Within the transition from trainee to independent practitioner, individuals may be well prepared for clinical work, but competence in managerial roles, service delivery, supervising residents and people management—areas important to the workplace culture and environment (including capacity for learning)—may be lacking [5, 9, 49–51]. The need for balance between supervision and opportunities for progressive independence, and the challenge of finding the right amount of each, has to be negotiated by supervisors and trainees on an individual basis. Less experienced supervisors may be more likely to be deterred by their lack of contextual experience, and therefore risk aversion on the part of the supervisor may adversely limit situational experience for postgraduate trainees.

#### Don’t knows

##### **Guideline 3.**

*What is the best balance between prescriptive programs tailored to individuals and ‘one size fits all’ competency-based or target-based training programs?*

Published evidence regarding the specialty-specific competencies required for individual specialties was deliberately excluded from this review. This heterogeneous body of literature currently shares a focus on what specialists in each area are believed to have mastered, rather than how the transition to independent specialist practice might be achieved. However, we did review evidence regarding generic medical knowledge, skills and behaviours. It is clear that it is not known what is best for learners regarding flexibility in training time to achieve competencies. Likewise, best practice in generic clinical and non-clinical training has not yet been identified. An example is that we do not know the best ways of assessing effectiveness in managing medical emergencies. Many training programs use simulation for this; however, there is insufficient literature available to come to a consensus on how to best assess effectiveness in the management of medical emergencies within the real healthcare setting. This is an area of great concern to newly qualified doctors as they make the transition into practice [52].

Although clinicians may well learn from significant events through reflection, there is currently insufficient evidence to comment upon whether this reflection on significant events is helpful to workplace-based learning in transition periods.

### Independence and responsibility

#### Do’s

##### **Guideline 4.**

*Provide meaningful pre-transition preparation courses linked to local inductions (* **moderate** *)*

Research on pre-transition training courses is almost exclusively related to the undergraduate to postgraduate transition. Preparation courses have become increasingly popular at the end of medical school, particularly following policy directives (e. g. from the General Medical Council in the UK). Perhaps as a result of this, many solely focus on preparation for specific medical skills and ignore the social, contextual, and psychological aspects of the imminent transition. Some of these courses are underpinned by short-term local evaluations in which student perceptions are commonly positive. The transition into being responsible for patient care may be less stressful as a result of these experiences, but it is not clear whether confidence correlates well to competence. Furthermore, following participants for extended periods of time identifies a waning of benefit which may become unidentifiable after a few months [53]. This suggests that impact on confidence may be greater than impact on actual competence. We lack high-quality studies of the correlation (or otherwise) between confidence and competence, and well-designed studies of impact of pre-transition training on outcomes other than subjects’ perceptions.

Experienced residents and consultants perceive less benefit from pre-transition training courses than students/postgraduate trainees [48, 54] and reporting of preparedness changes over time as postgraduate trainees gain experience and/or develop recall bias [4]. Effects of interventions may also be influenced by the personality traits of individual postgraduate trainees. Cave et al. [55] found that, as expected, doctors scoring highly on personality traits of conscientiousness and extraversion felt better prepared than those scoring highly for neuroticism. Therefore, it is probably valuable to improve the confidence of more ‘neurotic’ novices as their performance may be improved by reducing their anxiety levels [7, 27, 56].

The ability to endure uncertainty in new situations is protective and hence preparing new doctors to manage uncertainty seems logical [57]. Well-received preparations include discussing critical incidents [7, 58], providing career advice [59] as well as clinical aspects of work, such as shadowing opportunities [32, 33, 60]. The benefit of shadowing in a new workplace, alongside clinical skills training, is reported to reduce concerns about commencing work (such as managing acutely ill or dying patients, prescribing, and level of responsibility [4, 40]). For truly effective shadowing experiences, students and postgraduate trainees need to be able to learn on-the-job through having an allocated role in the team for supervised practice of the duties and responsibilities expected when they move to the next level of seniority or responsibility [4, 31, 43, 44, 51, 61]. Importantly, an emphasis on the difference between a stage of learning with patients (but carrying no responsibility for their management) and a stage of learning which involves direct responsibility for patient care, is not yet commonplace.

It is unusual for newly qualified doctors to receive deliberate on-the-job training in clinical reasoning, or more generally in how to manage complex cases [40, 62]. Meaningful methods of enhancing skills in administrative tasks, handovers, and prescribing [43, 63] are required (although the detail of what methods are most effective and efficient remains unknown). Additionally, time management may be crucial: one study identified it as a sensitive indicator of borderline postgraduate trainees (regardless of the other underlying challenges they might be facing [64]).

Much of the variance in perceptions within trainee groups and between postgraduate trainees and others remains unexplained [55]. Therefore, caution must be expressed regarding the effect of courses and shadowing experiences; there is uncertainty about what works best. However, given there are no studies suggesting preparation courses cause harm, even if all they achieve is a short-term reduction in stress, they have some benefit for those transitioning to independent practice.

##### **Guideline 5.**

*Encourage progressive independence by offering a sliding scale of decreasing supervision alongside demonstrating increasing trust (both globally and for specific tasks) (* **moderate** *)*

Progressive independence is grown through exposing learners to the ‘right’ mixture of challenge and support. What is ‘right’ is a matter of judgement and negotiation between the supervisor and trainee [4, 65, 66]. Benchmarks may be set as expectations (or in some cases, summative assessments) at particular stages of training but it is important that learners also know what is expected within a period of training (e. g. the years of residency) rather than just at the beginning or at the end.

Practical suggestions to support progressive learner independence include sharing constructive feedback between supervisors—perhaps in the form of work-based assessments—and establishing graduated levels of responsibility with which postgraduate trainees will be trusted as a result of particular levels of experience and performance [62].

Expertise development as an indicator of readiness for independent practice involves developing skills such as knowing when to apply ‘the usual routine’ to solve problems and when to draw on non-routine problem solving [67]. Over time postgraduate trainees will build a bank of experience from which they can create illness scripts [62] but they also need to know when there is a crucial deviation from one of these illness scripts. Engagement in progressive problem-solving and inclusion in non-routine complex case management is needed to ensure postgraduate trainees do not develop rigid thinking about illness scripts and thus case management [67].

##### **Guideline 6.**

*Apply the concepts of graduated responsibility to non-clinical as well as clinical domains of training, such as leadership and responsibility (* **moderate** *)*

Numerous issues relate to the transition to, and readiness to be, a consultant and many are non-clinical skills such as management and leadership. Unpreparedness within these domains is a significant source of concern and uncertainty [5, 11, 12, 49–51]. The apparent shock of discovering how much of a new role was taken up with activities away from direct patient contact can lead to a questioning of identity, which is also identified as a concern at undergraduate to postgraduate level [68]. Commitment to the specialty or department, as well as significant anxiety regarding practical issues such as financial management of specific services [5, 45] and transitions from trainee to consultant [12, 51] are prominent stressors. As a result, exposure and training should be designed to support progressive independence within both the clinical role *and* the essential non-clinical roles required in practice.

Brown et al. suggest that the transition phase into consultancy, if defined as full adjustment to new responsibilities, should be measured in years [5]. Adjustment and success are dependent not simply on new exposure to responsibility and different challenges, but also on how individuals act on their perceptions of these challenges and how they navigate change and novelty [66]. There is some evidence to suggest that contextual factors such as strong employment regulations and previous exposure to frameworks of progressive independence and social support may be protective against burnout during the transition to consultant [12, 13]. In another study by Brown et al. [6], educational supervisors of new pre-registration house officers said that only half had received training in how to be an educational supervisor, thereby identifying that exposure to supervision is not common practice, notwithstanding its relevance and significance.

##### **Guideline 7.**

*Make postgraduate trainees aware of the psychological impact of actual responsibility (including the process of their own identity formation) once they move up a level of training or into consultancy (* **moderate** *)*

Perceptions of competence are determined by different types of knowledge including: feedback received, volume of experience gained, awareness of limitations, observations of others, formal testing, self-assessment and awareness of complexity [14]. In contrast, sociocultural factors are the main determinants of independent function in practice; these factors include confidence, being judged as safe by others, case-specific clinical judgements, and differences in professional functioning according to time of day (with correlated staffing levels during out-of-hours shifts versus normal working [14]). The issues described above (Guideline 4: pre-transition courses) highlight the importance of the difference between perceptions of competence and determinants of independent function with respect to the transition from undergraduate to postgraduate practice. Brown et al. [5] identified that a significant change in colleagues’ perceptions about an individual who was moving from trainee to new consultant was often a source of stress to that individual, rather than being perceived as positive endorsement [12, 51]. Equally disconcerting, and with potential for negative impact on personal development, was the perception of new consultants that they might still be viewed as a resident [5].

#### Don’ts

##### **Guideline 8.**

*Treat transition as a moment in time (* **strong** *)*

The Oxford English Dictionary defines transition as ‘the process or a period of changing from one state or condition to another’; medical employment rarely offers formal space for transitions. Preparation, by definition, comes prior to responsibility and full integration into a role and hence time is necessary but not sufficient for good transitions [10, 36]. Once in role, doctors are expected to perform. There was no theoretical or empirical literature to support the idea that transition should be a moment in time. Rather, as argued by Teunissen [1], ‘situations lead to personal experiences … strings of experiences lead to multiple trajectories … reifications arise from recurrent activities’. All are required to complete a transition into independent practice [28] and the best chance of successful transition is when incremental autonomy in authentic practice has been well supported [14, 36, 39, 67].

#### Don’t knows

##### **Guideline 9.**

*What is the best way to ensure ongoing continuing professional development as a consultant or general practitioner?*

Further research is needed on ways to ensure effective ongoing development beyond graduation, board approval, or appointment as a consultant or general practitioner.

##### **Guideline 10.**

*Can we move understanding of the outcomes and impact of transition beyond perceptions?*

It is noteworthy that the vast majority of studies report perceptions of transition with a lack of research regarding alternative outcomes and impact. We postulate that this might have influenced the existent literature on the concept of transitions and therefore progressive independence.

##### **Guideline 11.**

*What is best practice in helping doctors-in-difficulty or training-departments-in-difficulty? In optimizing transitions what should be the role of regulatory bodies and employers?*

No studies were identified regarding this issue in relation to transitions. The movement of postgraduate trainees or consultants in difficulty between training settings, supervisors, and even more so between levels of seniority, is a significant concern as it is anticipated that the issues described in the rest of these guidelines would have even greater potential for adverse effects. Poorly functioning training departments and the impact of these on transition is another area in need of research. Research regarding the possible roles of regulators or human resources departments on postgraduate trainees or doctors in transition is also lacking.

### Mentoring & coaching support

#### Do’s

##### **Guideline 12.**

*Establish a mentorship program with local champions (* **moderate** *)*

We identified limited evidence regarding the role of mentorship in transitions. It is known that support from approachable and available supervisors capable of giving effective feedback, articulate expectations clearly, and function as good role models and teachers is important [7, 14]. Supervisors can provide effective mentorship for learners as they progress through individual rotations as well as their training program [7]. Failure of supervision impacts on the well-being of learners [7, 44]. Faculty development promotes and enhances supervisory skills [13]. Some authors have identified the importance of positive learning environments, team relationships and effective feedback in developing learners’ competence and confidence, which enhances postgraduate trainees’ perceptions of the safety of their own practice [14, 23, 44, 69]. All of these elements are important parts of supervision in the clinical workplace but some are also important components of effective workplace-based mentoring relationships.

Mentorship programs should seek to support the career of the mentee. Important criteria for a successful mentor are the ability to make time for postgraduate trainees [70], to be a good professional role model [71], and to be supportive while being able to provide appropriate challenging and constructive feedback [72]. Mentorship is seen as a major factor in achieving a successful career in academic medicine [73]. Career satisfaction, publication rate, retention, ability to progress were found to be improved by early and mid-career mentorship programs [74]. Straus identified that in order to be successful, the relationship between mentor and mentee must involve mutual respect and communication, the mentor must be skilled in the role, and there must be institutional support for and recognition of the value of mentoring as a professional activity [74]. It has been advocated that a formal mentorship program or support system should back up informal support systems, which are highly valued by some postgraduate trainees [5] and may be heavily relied upon in transitions. Although informal mentoring may work for those with ample informal support, this approach may prove insufficient for those who lack such support because of a new working environment or a lack of relationships within their workplace [5, 12]. Isolation in new workplaces leading to loneliness, and lack of mentorship during transitions have both been identified [75] as reasons for increased rates of burnout in the transition from trainee to consultant [51]. Confidence in practice comes with development of emotional competencies [76], the presence of good institutional support [7, 51] and supported reflection [8]. Good mentoring relationships provide a safe, supportive forum to discuss and develop these aspects of practice.

Within the transition to consultant, mentors are able to foster the development of skills in the managerial, practical and administrative aspects of practice as well as clinical skills [49, 50]. Reflection upon experiences, and peer discussion are both important in the socialization process of becoming an independent practitioner, and can take place within mentoring relationships [1, 65, 77, 78].

##### **Guideline 13.**

*Seek to aid the development of resilience and independence (* **moderate** *)*

Effective self-care, socialization, and learning ‘the system’ can all assist in adaptation to both a new role and a new system at the trainee level [7]. Such adaptations are needed frequently during training years and require resilience both to personal losses involved in moving from one role and location, to another, and the effort needed to build new relationships and learn new systems. Resiliency during transitions is an area in which further research may be forthcoming as its importance is more generally emphasized in healthcare literature. The concept of ‘resiliency’ was initially described by Hobfoil [79] in the *Conservation of Resources* theory. This theory describes stress models related to the loss of valued resources (which could be relationships, familiarity or a sense of competence). The theory’s two principles are first, that resource loss is more acutely felt then resource gain and second that one needs to invest in resources to regain resources. A person with more resources (such as self-confidence, knowledge, communication skills or a very supportive mentor) may have more ability to recover from a loss.

It should not be assumed that all learners will have the insight into how they can adapt to new environments and expectations. Thus, we can postulate that some, at least, might be assisted by sharing reflective and mindful approaches to learning as would be found in a mentoring relationship. In the transition to consultant, Wilkie and Raffaelli identify self-doubt as being common and describe a ‘fundamental re-examination of who and what we are’ [10]. Stresses that occur in this transition to consultancy can ease over time especially in a supportive work environment with the development of resilience and independence.

#### Don’t knows

##### **Guideline 14.**

*How do we develop common understanding around what is optimal in mentoring and coaching for multidimensional medical roles?*

Mentoring and coaching share some common activities and goals but are generally considered different in orientation. Mentorship often takes a global perspective on development and uses interpersonal relationships for reflection, while coaching (especially in sports) usually occurs over a shorter period of time and is more task driven, and aimed at achieving specific objectives. This means that mentorship does not usually involve active participation in assessment while professional coaching does include evaluation of performance in order to craft strategies for improvement [80].

The best format for mentorship in medicine is not known. Some have suggested personal matching programs for mentorship to be highly effective [80] but these are potentially costly and not always practical to arrange. Other studies recommend that potential mentees are taught strategies for identifying effective mentors rather than being assigned to a particular person [5, 72]. Therefore, the most effective format for setting up and delivering mentorship or coaching within medicine remains unknown.

For medical school graduates, role models are considered important in development of good clinical practice [55]. However, unlike formal mentors, role models are not part of the overt provision of support and they may have no awareness that they are a source of ‘guidance’, therefore they may not fully meet the learner’s needs from the perspective of supporting transitions to independent practice.

Passi et al. [81] highlight the need for learners to be educated in appraising the models they observe so that they can understand and reflect on negative role modelling and use it as an effective learning experience. Three characteristics of good role models are identified as clinical competence, teaching skills and personal qualities [82].

The lack of feedback for new consultants may lead to uncertainty in self [5] and identity [10].  A tolerance of uncertainty when first in consultant practice may be a critical skill but we do not know how best to train for it. It is not clear whether support and feedback should come from a mentor or from a more formal relationship such as the chief of a department or division. Brown et al. [5] suggest that the best environment for a new consultant is one in which many informal support structures are available and there is an ‘open door’ policy for advice. However, how to accomplish this remains unclear.

Team mentorship is another concept that has not been examined in detail but has been suggested as a possible solution. The efficacy of this is not known [72]. We also do not know whether video- or tele-mentoring is useful except for procedural-related skill enhancement, although there are increasing initiatives to offer these in large countries such as Canada with dispersed medical communities [83].

### Patient perspectives

Current evidence does not permit the formation of Do’s and Don’ts guidance with respect to patient perspectives from studies with patient participants or reporting patient concerns. We have retained an independent section to report the gaps (Don’t knows) most clearly identified in our scoping review regarding patient experience of professional transitions and the impact of these on patient care for two reasons. The first is methodological as explained in the methods section above. The second is that we, the authors, were collectively of the view that these gaps were a significant negative finding; a lack of patient perspective is pertinent to patients receiving care, particularly during times of mass professional transitions into new roles and levels of seniority and responsibility. This view is confirmed by a study of the stages of transition published just as we were revising these guidelines (hence not included in the scoping review) which demonstrates the patient-orientated concerns of medical students during the transition to being newly qualified doctors. Their concerns are also noted to reflect those of patients receiving healthcare in many settings [52].

#### Don’t knows

##### **Guideline 15.**

*How can patient feedback encourage effective transitions to independent practice and contribute to risk management?*

One might consider the patient perspective on transitions to independent practice as fundamental to ensuring meaningful benchmarks. Furthermore, patients might legitimately claim involvement to ensure progress is appropriately measured against such benchmarks. Patient-oriented concerns are likely to include both the specific (is this doctor able to provide a specific, possibly technical, intervention independently?) and global (should this doctor be leading my care?). Despite these arguments, none of the identified literature included patient participants or perspectives on their role in transitions to independent practice. It is possible that there are other bodies of literature, not linked to medical education research, covering patient views. However, it is also quite likely that, for historical, social, and academic reasons, there has been a divide between research seeking to understand learner progression, and research seeking to understand patient outcomes and impact. This might result from the challenges of demonstrating that educational interventions have a direct impact on patients, but also in part attributable to the divide that has arisen over time between research conducted into clinical training, and research conducted into patient outcomes.

##### **Guideline 16.**

*What is the best way to manage the tension between creating opportunities for progressive clinical independence as a learning mechanism and managing patient safety?*

The tension between the provision of safe and high-quality healthcare to patients, while maximizing opportunities for workplace learning, was featured in three papers. Patient safety guidelines present a major challenge to resolving this tension, although with adequate supervision the two aims are not mutually exclusive [4]. Kennedy et al. [46] derived a conceptual model from observation and interview data in large teaching hospitals accounting for the relationship between supervision and safety. The model describes different types of oversight employed by supervisors including identifying when they switch from oversight to direct intervention to deliver patient care themselves. While considering patient impact, this study did not include patients as participants and so the model relies on professional assumptions regarding the patient perspective. De Feijter et al.’s [47] study highlights the problems with this assumption. Their focus groups with final year medical students identified tensions and contradictions in the perceptions of students regarding what they needed to do to become doctors and what was required to deliver safe patient care. In particular, students held divergent views on what the boundaries of their responsibilities were according to the burden of perceived risk to patients weighed against the perceived need to learn or demonstrate learning. Together these studies point up an important area for further research. The needs are (i) to identify more clearly the extent to which patients are prepared to see immediate patient comfort potentially compromised, in order that clinician learning and competence can be developed and tested, and (ii) to better conceptualize patient safety, using patient and professional perspectives to balance safe care now and sustainable safe care in the future through learning and training.

## Conclusion

Initially the running title of this paper was ‘Supporting transition to independent practice’. However, as the work progressed, we realized that an objective to develop fully independent practitioners is probably unachievable, outdated, and undesirable. What is important for modern healthcare practice is recognition that transition is a continuum and that the need to mark stages in which physician-learners take on increasing responsibility introduces cyclical steps into what could otherwise be viewed as linear progression.

**Fig. 2:**
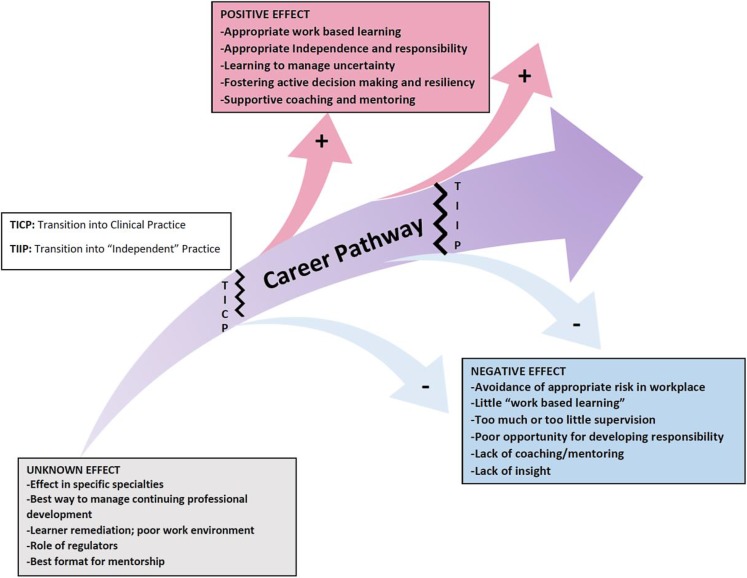
Development of progressive independence

Nonetheless, a conceptual notion of independent practice is important if only to demarcate consultant-level working and to incentivize learners. In this paper, we propose evidence-based guidelines aimed at facilitating transitions through the fostering of progressive independence (see Fig. 2). We consider our multidisciplinary collaboration, and combining of scoping review methods with expert consensus to be a strength of our work. Regarding limitations, it remains possible that a full systematically conducted review with greater resources might have identified further evidence. Inevitably there are areas (perhaps potential subjects for other D3 guides) that we have only been able to touch on because much of the evidence about specific pedagogical and other aspects of medical education is self-evidently pertinent in times of transition, but not necessarily different in transition to other times. The transition to independent practice is a global concept that depends on feedback [62] and assessment practices, requirements of regulatory bodies and the supervisor/supervisee relationship.

We hope that both learners and supervisors will keep in mind that total independence is illusory. Today’s physicians who work in teams, need to be co-dependent, not independent. They will continue to be supervised both by regulators and by peers, whose duty to draw attention to a colleague’s learning needs is increasingly enshrined in regulation, and patients’ feedback will feature ever more strongly in their ongoing development. Even the most experienced consultants will face future transitions as they seek to master new procedures or approaches. Thus, these guidelines are intended to assist the supervisory process of transitions of the ‘work-in-progress’ variety rather than encouraging a ‘job-done’ approach, hence our revised title ‘Supporting transition to more independent practice’. The strongest message from current evidence is that we should not view transitions as a moment in time, but opportunities for valuable personal and professional development which need to be supported.
